# THE INTERSECTION OF RELIGION AND SES IN MANAGING CHRONIC CONDITIONS AMONG OLDER PERSONS IN NIGERIA

**DOI:** 10.1093/geroni/igz038.2501

**Published:** 2019-11-08

**Authors:** Kafayat O Mahmoud

**Affiliations:** University of Kansas, Lawrence, Kansas, United States

## Abstract

Increased life expectancy in Nigeria has corresponded with higher rates of chronic diseases among older persons. Consequently, this is a new experience that older persons progressively have to deal with. In this study, I explored how religion and social support helped older persons cope with their chronic disease conditions, in light of the prevailing socio-cultural and economic circumstances in Nigeria. The research was conducted in two state-owned medical institutions, in a city in the North-Central part of Nigeria. In-depth, qualitative interviews were conducted among 19 purposively selected chronically ill persons aged between 50 years and over, during clinic days. The study revealed that religion is central to peoples’ management of feelings of despair, and acceptance of chronic disease conditions, as well as their adherence to prescriptions. This is explained by the theme “God as the Bestower and Reliever.” Also, some respondents perceived their coreligionists to be financially supportive. Although, some participants expressed that they depended on their families for their upkeep and emotional well-being, dire socio-economic conditions and lack of governmental support in chronic care meant that financial support was limited. This is explained by the theme “Times are Hard.” Subsequently, most respondents bore a dual burden of coping with chronic conditions even as they were financially responsible for themselves and their families. This was particularly stressful because it meant that most respondents were constantly worried about being able to meet basic daily needs, as well as manage the financial costs of their treatments, which proved expensive to manage.

